# Necrotizing soft tissue infection of the fingertips secondary to paronychia in a leukemic child: a case report and warning

**DOI:** 10.3389/fped.2025.1706507

**Published:** 2026-02-02

**Authors:** Nannan Li, Zhe Zhang, Yujie Lu, Huanlin Wu, Yu Zhang, Fenghui Cong

**Affiliations:** Da Lian Women and Children Medicine Group, Da Lian, China

**Keywords:** acute lymphoblastic leukemia, chemotherapy, multistep nursing, necrotizing soft tissue infection, paronychia

## Abstract

Acute lymphoblastic leukemia (ALL) is the most common childhood malignancy, for which chemotherapy remains the cornerstone of treatment. Skin toxicity is a frequent adverse reaction to chemotherapy; however, progression from paronychia to necrotizing soft tissue infection of the fingertips is uncommon. This report describes the case of a 7-year-old child with ALL who developed paronychia following combination chemotherapy with vincristine and daunorubicin. The infection progressed to involve multiple fingertips as necrotizing soft tissue infection, a course facilitated by the patient's underlying severe neutropenia. *Raoultella ornithinolytica* was identified on blood culture. A structured, multistep nursing protocol was implemented, including local disinfection, ethacridine lactate compresses, topical application of recombinant human epidermal growth factor solution and mupirocin ointment, and local oxygen insufflation. This regimen resulted in significant improvement of the lesions. Within 7 days, pain subsided, edema reduced by more than 50%, and joint mobility was largely restored, with complete clinical resolution achieved within 14 days. This case highlights the importance of early recognition of chemotherapy-induced skin toxicity and the value of standardized stepwise wound management in improving functional outcomes. It provides a practical reference for managing this rare yet severe cutaneous adverse reaction in children and underscores the critical role of specialized nursing in supportive oncology care.

## 1 Introduction

Acute lymphoblastic leukemia (ALL) is the most common pediatric malignancy, accounting for approximately 23% of all cancer diagnoses in patients under 15 years of age (1, 2). The management of pediatric ALL typically involves a multimodal strategy integrating chemotherapy, radiotherapy, and in selected cases, surgery. Systemic pharmacotherapy forms the cornerstone of treatment and primarily includes cytotoxic agents, corticosteroids (hormonal therapy), and molecularly targeted drugs, all aimed at inhibiting malignant cell proliferation (3). These drugs serve as indispensable components across all phases of therapy, including induction, consolidation, and maintenance.

However, antineoplastic agents are associated with a spectrum of adverse events (AEs). Nail toxicity is a well-documented cutaneous adverse effect associated with both conventional chemotherapeutic agents and novel targeted therapies (4). This susceptibility arises from the continuously proliferating nail matrix cells, which are highly vulnerable to chemotherapeutic damage. Clinical manifestations may involve three anatomical regions: the nail matrix (presenting as Beau's lines, onychomadesis, or melanonychia), the nail bed (resulting in onycholysis, subungual hemorrhage, or hematoma), and the proximal nail fold (including paronychia or periungual pyogenic granuloma) (5, 6). Nail growth is faster in children and adolescents, proceeding at an estimated rate of 0.12 mm/day (7, 8). Numerous chemotherapeutic agents and regimens have been demonstrated to induce nail changes, including taxoids, cyclophosphamide, doxorubicin/daunorubicin, 5-fluorouracil, and vincristine (9–12). Although not typically life-threatening, chemotherapy-induced nail changes can cause substantial cosmetic concerns, pain, and functional impairments, thereby adversely affecting quality of life and limiting daily and self-care activities (4, 13). Therefore, early and proactive management of these changes is necessary to preserve patient function and quality of life.

In this study, we report a case of a 7-year-old child with ALL who developed paronychia following combination chemotherapy with vincristine and daunorubicin. The condition progressed rapidly to necrotizing soft tissue infection. A structured wound care protocol was implemented, leading to a favorable clinical outcome. This case offers valuable insights into the evidence-based management of chemotherapy-induced paronychia complicated by necrotizing soft tissue infection and may help guide the optimization of clinical strategies for this complication among pediatric oncology patients.

## 2 Patient presentation

**Figure 1:**
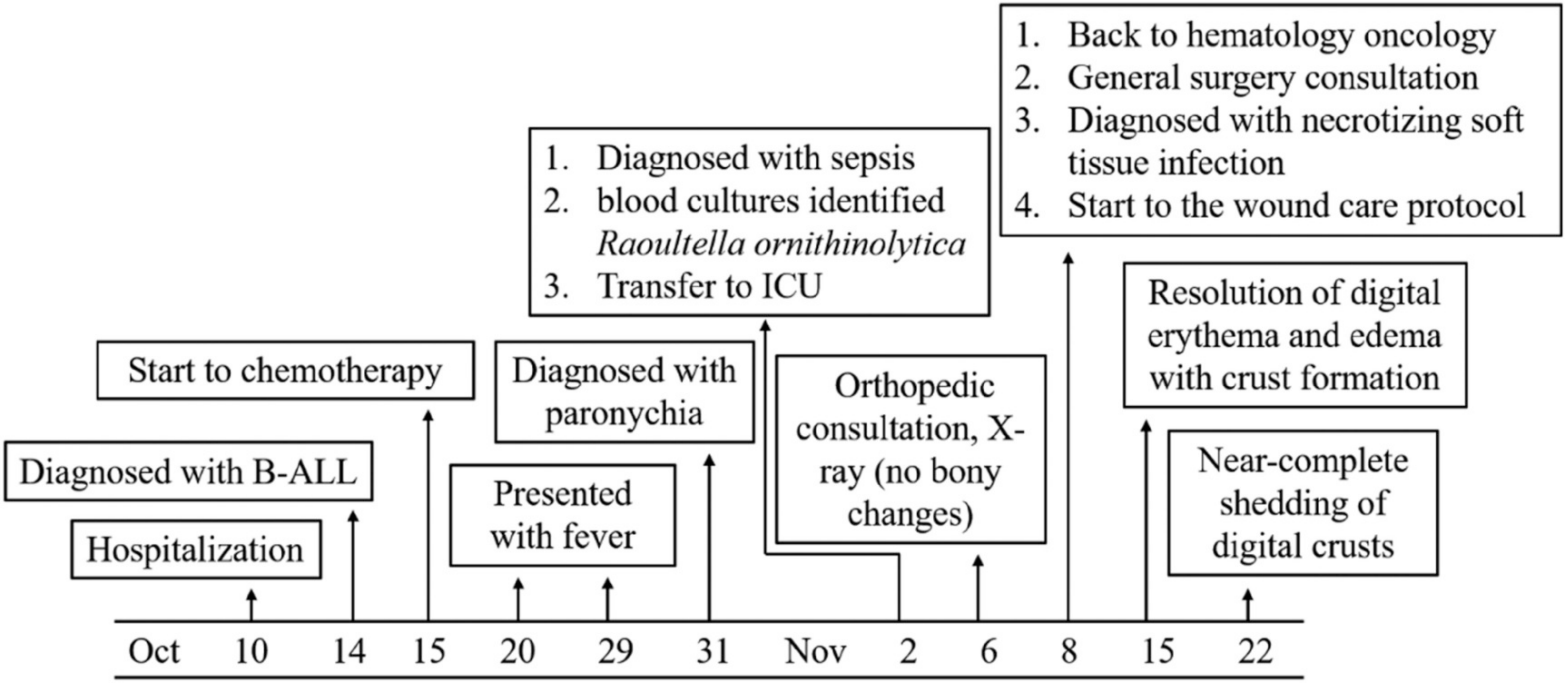
A clinical timeline of the pediatric patient.

A 7-year-old girl with no significant family history of hematological disorders, although with a documented digit-sucking habit, presented to our hospital with arthralgia and joint swelling lasting over one month, skin petechiae for three days, and abnormal blood test results for 3 h. The clinical timeline and laboratory data of the entire case are summarized in Figure 1 and Table 1. Initial laboratory findings revealed thrombocytopenia (platelet count: 12 × 10⁹/L) and neutropenia (absolute neutrophil count: 0.79 × 10⁹/L), prompting hospitalization with a provisional diagnosis of immune thrombocytopenic purpura. Bone marrow aspiration cytology on day 3 confirmed acute leukemia, and subsequent immunophenotyping established a definitive diagnosis of B-cell acute lymphoblastic leukemia (B-ALL). According to the *Chinese Children's Cancer Group* 2020 protocol (CCCG-ALL-2020), the patient was classified into the intermediate-/high-risk group and received the corresponding remission induction chemotherapy. The specific regimen consisted of the following: an initial 4-day intravenous course of dexamethasone (6 mg/m^2^, q8h); from day 5 onward, daily oral prednisone (60 mg/m^2^, q8h) combined with dexamethasone (8 mg/m^2^, q8h), plus single intravenous doses of vincristine (1.5 mg/m^2^) and daunorubicin (25 mg/m^2^) on day 5 and on day 6, and a single intramuscular injection of pegaspargase (2,000 U/m^2^) along with triple intrathecal therapy (methotrexate 12.5 mg, cytarabine 35 mg, and dexamethasone 5.0 mg) for central nervous system leukemia prophylaxis. Transfusion support was provided in accordance with institutional guidelines to maintain platelets >20 × 10⁹/L and hemoglobin >80 g/L.

**Table 1:** The laboratory characteristic of the pediatric patient in the entire case.

Laboratory data	10.10	10.31	11.02	11.07	11.17	11.22
Routine blood test parameters						
WBC(×10^9^/L)	5.98	0.37	0.23	0.3	1.53	2.48
NEUT(×10^9^/L)	0.79	0.04	0.02	0.16	0.63	1.34
PLT (×10^9^/L)	12	23	13	48.00	43.00	324.00
PCT (%)	0.009	0.02	0.009	0.05	0.05	0.34
CRP(mg/L)	47.23	207.34	37.99	137.27	0.9	<0.80
Na^+^ (mmol/L)	131.0	131.1	132.3	133.8	136.5	137.6
Liver and kidney function parameters						
ALT(U/L)	25	30	40	54	48	22
AST(U/L)	46	22	32	33	27	22
LDH (U/L)	472	213	234	177	186	170
Cr (μmol/L)	40.2	26.0	22.4	19.2	23.3	27.8
BUN (μmol/L)	3.85	4.48	5.49	6.90	6.23	1.21
Coagulation parameters						
Prothrombin time	13.9	13.8	14.0	13.1	13.7	13.7
International normalized ratio	1.08	1.05	1.07	0.98	1.04	1.04
Activated partial thromboplastin time (s)	38.5	39.9	39.9	42.2	36.4	41.5
Thrombin time (s)	17.4	17.4	16.7	16.4	17.6	19.0
Fibrinogen	2.38	3.54	3.25	7.05	2.53	2.46
D-dimer (mg/L)	3.39	1.67	2.80	2.47	1.50	0.64
Prothrombin time activity percentage (%)	88	92	89	103	94	93

**Figure 2:**
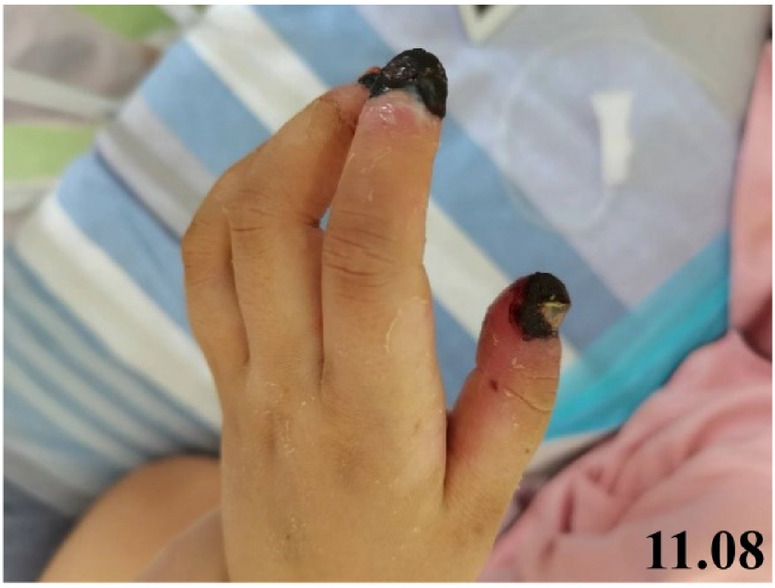
A clinical photograph taken during examination showing a necrotic lesion on the right fingertip. Informed consent for the publication of clinical images was obtained from the patient's parents.

During chemotherapy, the patient exhibited involuntary finger-picking behavior. She developed febrile episodes on days 10 and 19, with peak temperatures of 39.0 °C and 39.4 °C, respectively. On day 21, yellow purulent discharge appeared around the nails of the right hand, accompanied by tenderness but with preserved finger joint mobility. A diagnosis of paronychia was made, and topical ethacridine lactate solution was applied three times daily. By day 23, the patient had been diagnosed with sepsis following blood cultures identification of *Raoultella ornithinolytica*. On day 27, identification of the fingertips deteriorated, manifesting as ulceration, epidermal desquamation, and erythema. An orthopedic consultation and X-ray were performed, which revealed no evidence of bony abnormalities. Mupirocin ointment was added to the treatment regimen for enhanced infection control. By day 29, the patient had experienced a rapid worsening of the fingertip lesions, which progressed to dark purple discoloration accompanied by severe pain, indicating progression to necrotizing soft tissue infection. The evolution of digital clinical manifestations is summarized in Table 2. The clinical presentation was distinguished from purpura fulminans and ecthyma gangrenosum based on the observed features and supporting laboratory indicators (Table 1). A general surgery consultation was subsequently obtained. Given the absence of signs indicating deep tissue involvement, management proceeded with close observation. Subsequent pain and swelling led to flexion contractures of the fingers, severely impairing activities of daily living (Figure 2).

**Table 2:** Temporal progression and management of necrotizing fingertip infection.

Parameter	10.31	11.02	11.05	11.06	11.07	11.08	11.13	11.15	11.22
Department	Hematology/oncology			ICU		Hematology/Oncology			
Fingers involved	Right thumb, left thumb, and index finger			All except little fingers			All fingers		
Purulent discharge	Present								
Ulceration		Present	Present	Present	Present	Present			
Epidermal desquamation				Present	Present	Present			
Erythema				Present	progressed to dark purple discoloration	dark purple			
Edema					Present	Present	Present	Present	
Serous exudation					Present	Present	Present	Largely resolved	
Crust formation							Present	Present	Nearly complete shedding
Tenderness					Present	Present			
Fluctuance						Present	Largely resolved		
Digital mobility					Pain-induced complete immobility	Pain-induced complete immobility			
Management	Ethacridine lactate, q8h			Ethacridine lactate and mupirocin, q8h			The wound care protocol in the present study		

**Figure 3:**
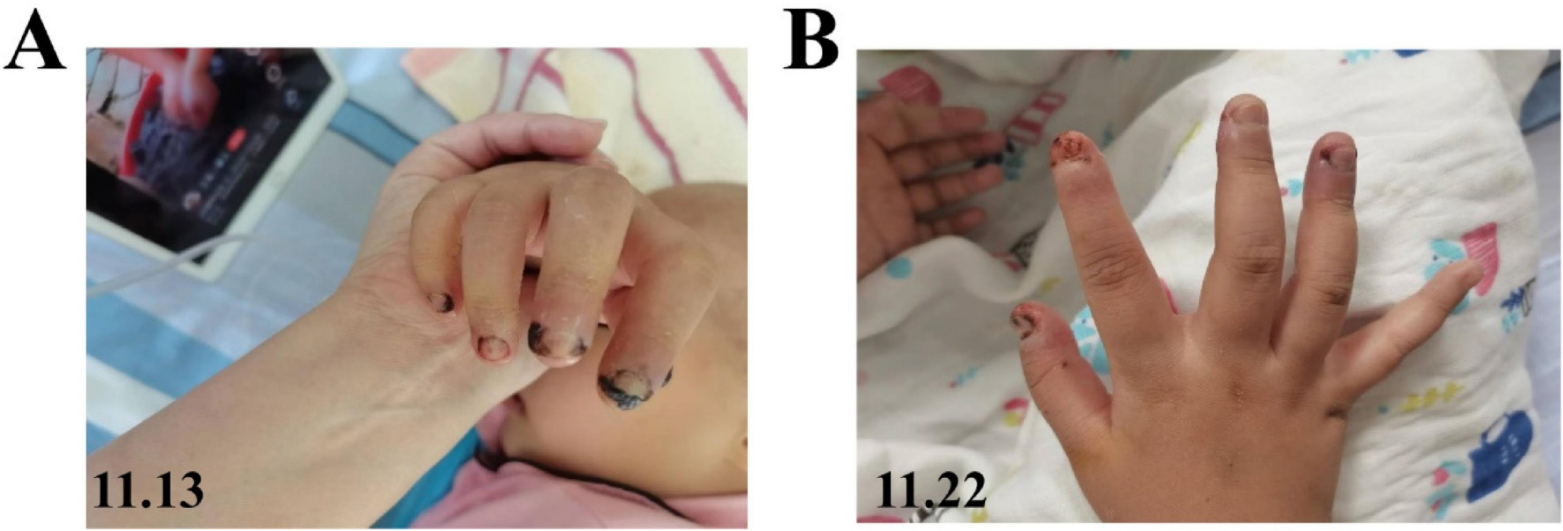
Findings from clinical photography demonstrating the progression of wound healing following 1 week **(A)** and 2 weeks **(B)** of comprehensive nursing intervention. Informed consent for the publication of clinical images was obtained from the patient's parents.

Following the diagnosis of necrotizing soft tissue infection, the child was administered intravenous meropenem (15 mg/kg, q8h). Concurrently, a series of targeted and systematic nursing interventions were initiated. The nursing protocol included the following: (1) systematic daily pharmacotherapeutic monitoring for potential adverse drug reactions such as rash, pruritus, and drug-induced fever; (2) patient and caregiver education to avoid hand contact with objects and refrain from scratching skin lesions; (3) a structured wound care protocol administered six times daily (two times each in the morning, afternoon, and evening) during the initial 4-day period. Each wound care session comprised the following: firstly, a 20-min application of 75% ethanol followed by 15–20-min compresses with ethacridine lactate solution compresses; then, topical administration of recombinant human epidermal growth factor solution (Jinyinte®) and mupirocin ointment (Bactroban®) to violaceous edematous areas; and finally, 10–20 min of local oxygen insufflation to enhance drug absorption. Supplemental lidocaine hydrochloride gel was applied as needed (maximum twice daily) for relief of paroxysmal pain, with oral acetaminophen reserved for breakthrough pain. The analgesic gel was discontinued on day 5 following pain resolution, while the core four-step care cycle was maintained. After 7 days of targeted care, violaceous edema had regressed by more than 50% with significant pain reduction. Partial functional recovery of finger extension was achieved, accompanied by a marked improvement in range of motion (Figure 3A). With continued application of the four-step core care, the violaceous edema and the associated inflammation were eliminated completely within 14 days (Figure 3B).

## 3 Discussion

Hematologic malignancies, which originate from the hematopoietic system, are highly prevalent in the pediatric population and pose a significant threat to both the physical and the psychological wellbeing of affected children (14). In China, the peak incidence of childhood cancer occurs between 1 and 4 years of age, with leukemia representing 32.89% and constituting the most common malignancy (15). Acute lymphoblastic leukemia (ALL) is the most frequent subtype of pediatric leukemia, accounting for approximately 70% of childhood leukemia cases and approximately 25% of all malignancies in children under 15 years of age. The annual incidence of ALL in China is 3–4 cases per 100,000 children, a rate roughly three times higher than that observed in adults (15).

The primary treatment modalities for pediatric ALL include chemotherapy, hematopoietic stem cell transplantation, targeted immunotherapy, and chimeric antigen receptor T-cell immunotherapy (CAR-T). Chemotherapy remains the foundational first-line approach (16, 17). In China, commonly used chemotherapeutic protocols include the CCLG-ALL 2018 and CCCG-ALL-2015/2020 regimens. At the international level, protocols from the Children's Oncology Group (COG) and the Berlin–Frankfurt–Munster (BFM) study group are widely adopted (18–21). Notably, the CCCG-ALL-2015/2020 regimen employs refined disease classification and minimal residual disease (MRD) monitoring to guide risk stratification and treatment planning, achieving a 5-year overall survival (OS) rate exceeding 95% in children with ALL (22). In clinical practice, the selection and adjustment of chemotherapy regimens are based on a comprehensive assessment of factors including leukemia subtype, genetic profile, individual drug metabolism, and treatment response. This personalized strategy aims to maximize therapeutic efficacy while minimizing toxicity (23). Treatment typically follows a multiphase structure. The induction phase, designed to achieve complete remission, frequently combines agents such as vincristine, prednisone, daunorubicin, and L-asparaginase to rapidly reduce the leukemic burden. This is followed by a consolidation phase, which often utilizes high-dose methotrexate and cytarabine. Subsequently, maintenance therapy administered for 2–3 years and usually consisting of low-dose methotrexate and 6-mercaptopurine is critical for preventing relapse (24–31).

The limitations of chemotherapy arise principally from the considerable toxicity of its agents. These drugs can produce a range of adverse effects that may disrupt treatment and, in severe cases, necessitate its interruption. Cutaneous toxicity is among the frequently observed adverse reactions, and nail toxicity represents a particularly notable manifestation (31–33). Investigation of chemotherapy-related nail changes is hindered by several confounding factors: first, the use of multiple agent regimens complicates the identification of a single causative drug; second, frequent modifications in therapy for progressing or refractory disease may lead to cumulative damage to the growing nails; and third, opportunistic infections occurring during chemotherapy, radiotherapy, or combined surgical treatment can also impair nail development (4). In the present case, the development of paronychia in the child was likely related to the concomitant administration of vincristine and daunorubicin.

The subsequent rapid deterioration of the condition was likely precipitated by the child's unconscious picking behavior, which breached the skin barrier and facilitated bacterial entry. Against a background of severe neutropenia, the infection became uncontrolled and progressed swiftly, culminating in abscess formation. The resulting increase in local tissue pressure further compressed the microvasculature, leading to tissue ischemia and necrosis that clinically manifested as dark purple edema of the fingertips. The diagnosis of necrotizing soft tissue infection in this case was primarily clinical, based on the rapidly progressive tissue edema, evolution from cyanosis to dark purple discoloration, severe pain, and systemic signs of infection in a neutropenic host. This clinical impression was supported by a LRINEC score ≥6 points (34, 35). The presentation was distinguished from purpura fulminans by normal coagulation parameters and the absence of disseminated intravascular coagulation (Table 1) (36). The coagulation profile, characterized by persistently elevated D-dimer and a single episode of raised fibrinogen, was primarily attributable to inflammatory and coagulant activation secondary to systemic sepsis. Furthermore, the clinical picture differed markedly from ecthyma gangrenosum, which typically presents as painless macules or punched-out ulcers with a central eschar (37). This patient exhibited painful, diffuse swelling and cyanosis of the fingertips that progressed rapidly to necrosis, a presentation consistent with necrotizing infection in the setting of severe neutropenia and bacteremia. In this immunocompromised pediatric case, a chemotherapy-induced paronychia rapidly progressed to sepsis and necrotizing infection following minor local trauma. Severe complication aligns with the pathophysiological framework reviewed by Słonimska et al., wherein chemotherapeutic agents systemically impair tissue repair through multiple pathways, including the suppression of cellular proliferation, reduction of growth factor synthesis, compromise of immune responses, and disruption of angiogenesis (38). Consequently, even minor inflammatory lesions become highly susceptible to progression into extensive tissue necrosis, underscoring that such complications represent a systemic failure of wound healing. This underscores that clinical management must integrate early anti-infective therapy, meticulous wound care, and systemic support, alongside investigating targeted strategies to promote tissue repair.

Raoultella species, belonging to the Enterobacteriaceae family, are gram-negative, aerobic, opportunistic pathogens commonly encountered in immunocompromised hosts, including cancer patients. Typical clinical presentations include biliary tract infections, pneumonia, and bacteremia (39–41). In contrast to adults, pediatric infections are primarily bloodstream-derived (42). Epidemiological data indicate that *Raoultella ornithinolytica* predominantly infects children over 3 years of age and newborns, with no significant gender predilection. Underlying conditions in older children frequently involve malignancies or immune disorders, a finding consistent with a report by Seng et al., in which approximately half of 112 pediatric cases had tumor- or immune-related diseases (43). *R. ornithinolytica* exhibits intrinsic resistance to ampicillin mediated by β-lactamase production. Current therapeutic evidence supports the use of cephalosporins, carbapenems, quinolones, and aminoglycosides as effective options (44–46). Although timely antibiotic intervention generally yields favorable outcomes, fatal cases have been reported (43). In the present case, blood culture confirmed the presence of *R. ornithinolytica* bacteremia. Meropenem, a carbapenem antibiotic that acts via irreversible inhibition of bacterial cell wall synthesis and is a cornerstone agent for infections caused by ESBL-producing Gram-negative bacilli, was selected for treatment (47). Treatment was administered for 10 days, resulting in resolution of fever and a reduction in inflammatory markers within 72 h.

The structured wound care protocol was designed based on institutional experience and supporting evidence from the literature. Ethacridine lactate compresses were chosen for their broad antiseptic action and low tissue toxicity, qualities that make them suitable for managing exudative, erosive, and contaminated wounds, including venous ulcers (48, 49). They are often used in debridement and dressing of various traumas, exudations, erosive infectious wounds, and skin lesions such as venous ulcers (50). Topical recombinant human epidermal growth factor (rh-EGF) was incorporated to promote re-epithelialization in necrotic tissue (51, 52). Mupirocin ointment was selected for its targeted activity against primary skin pathogens while preserving commensal flora, thereby supporting the skin's natural barrier function in both primary and secondary infections (53). The protocol also aimed to optimize the local wound environment. Lactate, in the presence of oxygen, stimulates fibroblast proliferation and angiogenesis (54). Since optimal epithelial regeneration occurs at oxygen concentrations between 10% and 50%, the regimen was designed to support this range (55–57). The potential adjunctive role of hyperbaric oxygen therapy in enhancing fibroblast function and keratinocyte differentiation was also considered within this physiological framework (58).

Systematic nursing care plays a crucial role in managing pediatric ALL patients who develop chemotherapy-induced paronychia that progress to necrotizing soft tissue infection. At present, no standardized, uniformly effective nursing protocol exists for this complication. In providing targeted care and pharmacovigilance, nursing staff should emphasize proactive communication with the patient and family, delivering psychological support and disease-specific health education. Fostering a positive and optimistic outlook toward treatment is essential to promote both psychological and physiological recovery. In the case of the patient in this study, the implementation of a structured wound-care protocol was associated with marked clinical improvement.

## 4 Conclusion

In conclusion, we report the successful management of a pediatric patient with ALL in whom chemotherapy-induced paronychia progressed rapidly to necrotizing soft tissue infection of the fingertips. The nursing team was instrumental in promptly implementing a structured, multistep wound care protocol. The protocol effectively controlled the infection, promoted tissue regeneration, and helped restore function despite the patient's profoundly immunocompromised state due to severe neutropenia. This case highlights the importance of early warning, systematic assessment, and standardized stepwise wound management in severe chemotherapy-related skin toxicity to improve clinical outcomes. Our experience provides valuable insights into managing rare, but critical adverse drug reactions in pediatric oncology nursing underscore the essential role of specialized nursing in supportive cancer care.

## Data Availability

The original contributions presented in the study are included in the article/Supplementary Material, and further inquiries can be directed to the corresponding author.

## References

[1] Jemal A Siegel R Ward E Murray T Xu J Smigal C Thun MJ. Cancer statistics, 2006. CA Cancer J Clin. (2026) 56(2):106–30. 10.3322/canjclin.56.2.106 16514137

[2] Gurieva OD Savelyeva MI Valiev TT Sozaeva ZA Kondratenko SN Ilyin MV. Pharmacogenetic aspects of efficacy and safety of methotrexate treatment in pediatric acute lymphoblastic leukemia. Drug Metab Pers Ther. (2023) 38(4):349–57. 10.1515/dmpt-2023-0079 38098143

[3] Zaiem A Hammamia SB Aouinti I Charfi O Ladhari W Kastalli S Hand-foot syndrome induced by chemotherapy drug: case series study and literature review. Indian J Pharmacol. (2022) 54(3):208–15. 10.4103/ijp.ijp_175_21 35848692 PMC9396690

[4] Chen W Yu YS Liu YH Sheen JM Hsiao CC. Nail changes associated with chemotherapy in children. J Eur Acad Dermatol Venereol. (2007) 21(2):186–90. 10.1111/j.1468-3083.2006.01887.x 17243953

[5] Daniel CR 3rd Scher RK. Nail changes secondary to systemic drugs or ingestants. J Am Acad Dermatol. (1984) 10(2 Pt 1):250–8. 10.1016/S0190-9622(84)70032-6 6371069

[6] Chen GY Chen YH Hsu MM Tsao CJ Chen WC. Onychomadesis and onycholysis associated with capecitabine. Br J Dermatol. (2001) 145(3):521–2. 10.1046/j.1365-2133.2001.04391.x 11531857

[7] Bean WB. Nail growth: 30 years of observation. Arch Intern Med. (1974) 134(3):497–502. 10.1001/archinte.1974.00320210107015 4137073

[8] Lavelle C. The effect of age on the rate of nail growth. J Gerontol. (1968) 23(4):557–9. 10.1093/geronj/23.4.557 5723495

[9] Minisini AM Tosti A Sobrero AF Mansutti M Piraccini BM Sacco C Taxane-induced nail changes: incidence, clinical presentation and outcome. Ann Oncol. (2003) 14(2):333–7. 10.1093/annonc/mdg050 12562663

[10] Rafi L Friedrich M Tilgen W Reichrath J. Severe nail changes due to docetaxel treatment. Eur J Dermatol. (2003) 13(6):610–1. 14721789

[11] Leonard GD Zujewski JA. Docetaxel-related skin, nail, and vascular toxicity. Ann Pharmacother. (2003) 37(1):148. 10.1345/aph.1C190 12564432

[12] Tinio P Bershad S Levitt JO. Medical pearl: docetaxel-induced onycholysis. J Am Acad Dermatol. (2005) 52(2):350–1. 10.1016/j.jaad.2004.07.057 15692485

[13] Piraccini BM Tosti A. Drug-induced nail disorders: incidence, management and prognosis. Drug Saf. (1999) 21(3):187–201. 10.2165/00002018-199921030-00004 10487397

[14] Yang WY Zhu XF. [Current situation and prospect of diagnosis and therapy of childhood acute leukemia in China]. Zhonghua Yi Xue Za Zhi. (2024) 104(27):2477–82. Chinese. 10.3760/cma.j.cn112137-20231211-01347 38978372

[15] Cronin KA Scott S Firth AU Sung H Henley SJ Sherman RL Annual report to the nation on the status of cancer, part 1: national cancer statistics. Cancer. (2022) 128(24):4251–84. 10.1002/cncr.34479 36301149 PMC10092838

[16] Malczewska M Kośmider K Bednarz K Ostapińska K Lejman M Zawitkowska J. Recent advances in treatment options for childhood acute lymphoblastic leukemia. Cancers (Basel). (2022) 14(8):2021. 10.3390/cancers14082021 35454927 PMC9032060

[17] Butler E Ludwig K Pacenta HL Klesse LJ Watt TC Laetsch TW. Recent progress in the treatment of cancer in children. CA Cancer J Clin. (2021) 71(4):315–32. 10.3322/caac.21665 33793968

[18] Liu X Zou Y Zhang L Guo Y Chen Y Yang W A novel risk defining system for pediatric T-cell acute lymphoblastic leukemia from CCCG-ALL-2015 group. Front Oncol. (2022) 12:841179. 10.3389/fonc.2022.841179 35296004 PMC8920043

[19] Brown P Inaba H Annesley C Beck J Colace S Dallas M Pediatric acute lymphoblastic leukemia, version 2.2020, NCCN clinical practice guidelines in oncology. J Natl Compr Canc Netw. (2020) 18(1):81–112. 10.6004/jnccn.2020.0001 31910389

[20] Zhang X Wang Y Tian X Sun L Jiang H Chu J Delineation of features, responses, outcomes, and prognostic factors in pediatric PDGFRB-positive acute lymphoblastic leukemia patients: a retrospective multicenter study. Cancer. (2024) 130(22):3902–12. 10.1002/cncr.35481 39136180

[21] Campbell M Kiss C Zimmermann M Riccheri C Kowalczyk J Felice MS Childhood acute lymphoblastic leukemia: results of the randomized acute lymphoblastic leukemia intercontinental-Berlin-Frankfurt-Münster 2009 trial. J Clin Oncol. (2023) 41(19):3499–511. 10.1200/JCO.22.01760 37141547

[22] Yang W Cai J Shen S Gao J Yu J Hu S Pulse therapy with vincristine and dexamethasone for childhood acute lymphoblastic leukaemia (CCCG-ALL-2015): an open-label, multicentre, randomised, phase 3, non-inferiority trial. Lancet Oncol. (2021) 22(9):1322–32. 10.1016/S1470-2045(21)00328-4. Erratum in: Lancet Oncol. 2021 22(9):e389. 34329606 PMC8416799

[23] Brown PA Shah B Advani A Aoun P Boyer MW Burke PW Acute lymphoblastic leukemia, version 2.2021, NCCN clinical practice guidelines in oncology. J Natl Compr Canc Netw. (2021) 19(9):1079–109. 10.6004/jnccn.2021.0042 34551384

[24] Ronghe M Burke GA Lowis SP Estlin EJ. Remission induction therapy for childhood acute lymphoblastic leukaemia: clinical and cellular pharmacology of vincristine, corticosteroids, L-asparaginase and anthracyclines. Cancer Treat Rev. (2001) 27(6):327–37. 10.1053/ctrv.2001.0243 11908926

[25] Capria S Molica M Mohamed S Bianchi S Moleti ML Trisolini SM A review of current induction strategies and emerging prognostic factors in the management of children and adolescents with acute lymphoblastic leukemia. Expert Rev Hematol. (2020) 13(7):755–69. 10.1080/17474086.2020.1770591 32419532

[26] Wolff SN Herzig RH Fay JW Phillips GL Lazarus HM Flexner JM High-dose cytarabine and daunorubicin as consolidation therapy for acute myeloid leukemia in first remission: long-term follow-up and results. J Clin Oncol. (1989) 7(9):1260–7. 10.1200/JCO.1989.7.9.1260 2769327

[27] Kim DS Kang KW Lee SR Park Y Sung HJ Kim SJ Comparison of consolidation strategies in acute myeloid leukemia: high-dose cytarabine alone versus intermediate-dose cytarabine combined with anthracyclines. Ann Hematol. (2015) 94(9):1485–92. 10.1007/s00277-015-2389-9 25944346

[28] Schmiegelow K Nielsen SN Frandsen TL Nersting J. Mercaptopurine/methotrexate maintenance therapy of childhood acute lymphoblastic leukemia: clinical facts and fiction. J Pediatr Hematol Oncol. (2014) 36(7):503–17. 10.1097/MPH.0000000000000206 24936744 PMC4222610

[29] Toksvang LN Als-Nielsen B Bacon C Bertasiute R Duarte X Escherich G Thiopurine enhanced ALL maintenance (TEAM): study protocol for a randomized study to evaluate the improvement in disease-free survival by adding very low dose 6-thioguanine to 6-mercaptopurine/methotrexate-based maintenance therapy in pediatric and adult patients (0–45 years) with newly diagnosed B-cell precursor or T-cell acute lymphoblastic leukemia treated according to the intermediate risk-high group of the ALLTogether1 protocol. BMC Cancer. (2022) 22(1):483. 10.1186/s12885-022-09522-3 35501736 PMC9063225

[30] Murphy T Yee KWL. Cytarabine and daunorubicin for the treatment of acute myeloid leukemia. Expert Opin Pharmacother. (2017) 18(16):1765–80. 10.1080/14656566.2017.1391216 29017371

[31] Pavey RA Kambil SM Bhat RM. Dermatological adverse reactions to cancer chemotherapy. Indian J Dermatol Venereol Leprol. (2015) 81(4):434. 10.4103/0378-6323.159950 26144855

[32] Özhan AK Demirhan A Arikoglu T Karahan F Satıcı FEG Tokmeci N Toxic skin reactions should be differentiated from allergic reactions to chemotherapeutic drugs in children: a case series and review of the literature. Dermatitis. (2024) 35(3):275–87. 10.1089/derm.2023.0258 38165639

[33] Agarwal A Panda SP Panda M Jena AK. Spectrum of dermatology referrals from the pediatric cancer unit: a prospective observational study. Indian J Paediatr Dermatol. (2025) 26(2):97–102. 10.4103/ijpd.ijpd_103_24

[34] Stevens DL Bryant AE Goldstein EJ. Necrotizing soft tissue infections. Infect Dis Clin North Am. (2021) 35(1):135–55. 10.1016/j.idc.2020.10.004 33303335

[35] Wong CH Khin LW Heng KS. The LRINEC (Laboratory Risk Indicator for Necrotizing Fasciitis) score: a tool for distinguishing necrotizing fasciitis from other soft tissue infections. Crit Care Med. (2004) 32(7):1535–41. 10.1097/01.CCM.0000129486.35458.7D 15241098

[36] Thrombosis and Hemostasis Group, Hematology Society of Chinese Medical Association. [Consensus of Chinese experts on diagnosis of disseminated intravascular coagulation (version 2017)]. Zhonghua Xue Ye Xue Za Zhi. (2017) 38(5):361–3. Chinese. 10.3760/cma.j.issn.0253-2727.2017.05.001 28565731 PMC7354191

[37] Gałązka P Kaczor P Kałużny K Leis K. Ecthyma gangrenosum as a serious complication of Pseudomonas aeruginosa infection in departments of paediatric oncology. Postepy Dermatol Alergol. (2021) 38(4):537–43. 10.5114/ada.2020.100747 34658690 PMC8501443

[38] Słonimska P Sachadyn P Zieliński J Skrzypski M Pikuła M. Chemotherapy-mediated complications of wound healing: an understudied side effect. Adv Wound Care (New Rochelle). (2024) 13(4):187–99. 10.1089/wound.2023.0097 38183626 PMC10924052

[39] Sękowska A. Raoultella spp.—clinical significance, infections and susceptibility to antibiotics. Folia Microbiol (Praha). (2017) 62(3):221–7. 10.1007/s12223-016-0490-7 28063019

[40] Camargo CH Yamada AY de Souza AR Sacchi CT Reis AD Santos MBN Genomic characterization of New Delhi metallo-beta-lactamase-producing species of Morganellaceae, Yersiniaceae, and Enterobacteriaceae (other than Klebsiella) from Brazil over 2013–2022. Microbiol Immunol. (2024) 68(1):1–5. 10.1111/1348-0421.13100 37859304

[41] Boukaka Kala RG Diatewa B Bingui Outman PD Nkounkou Banzouzi IC Lamah L Nkounkou Milandou Kadidia GC Méningite bactérienne néonatale à Raoultella planticola: à propos d’un cas. J Pédiatrie Puéric. (2023) 36(6):298–302. 10.1016/j.jpp.2023.05.001

[42] Haruki Y Hagiya H Sakuma A Murase T Sugiyama T Kondo S. Clinical characteristics of Raoultella ornithinolytica bacteremia: a case series and literature review. J Infect Chemother. (2014) 20(9):589–91. 10.1016/j.jiac.2014.05.005 25012469

[43] Seng P Boushab BM Romain F Gouriet F Bruder N Martin C Emerging role of Raoultella ornithinolytica in human infections: a series of cases and review of the literature. Int J Infect Dis. (2016) 45:65–71. 10.1016/j.ijid.2016.02.014 26921549

[44] Walckenaer E Poirel L Leflon-Guibout V Nordmann P Nicolas-Chanoine MH. Genetic and biochemical characterization of the chromosomal class A beta-lactamases of Raoultella (formerly Klebsiella) planticola and Raoultella ornithinolytica. Antimicrob Agents Chemother. (2004) 48(1):305–12. 10.1128/AAC.48.1.305-312.2004 14693555 PMC310189

[45] Hostacká A Klokocníková. Antibiotic susceptibility, serum response and surface properties of Klebsiella species. Microbios. (2001) 104(408):115–24. 11297012

[46] Pi DD Zhou F Bai K Liu C Xu F Li J. Raoultella ornithinolytica infection in the pediatric population: a retrospective study. Front Pediatr. (2020) 8:362. 10.3389/fped.2020.00362 32754562 PMC7366290

[47] Huang YS Zhou H. Breakthrough advances in beta-lactamase inhibitors: new synthesized compounds and mechanisms of action against drug-resistant bacteria. Pharmaceuticals (Basel). (2025) 18(2):206. 10.3390/ph18020206 40006020 PMC11859904

[48] Jabri T Khan NA Makhlouf Z Akbar N Gul J Shah MR Antibacterial properties of ethacridine lactate and sulfmethoxazole loaded functionalized graphene oxide nanocomposites. Antibiotics. (2023) 12:755. 10.3390/antibiotics12040755 37107117 PMC10135308

[49] Filipe Rosa L Gonda S Roese N Bischoff SC. Tannic acid and ethacridine lactate attenuate markers of stress-induced intestinal barrier dysfunctions in murine small intestinal organoids. Biomolecules. (2025) 15(5):650. 10.3390/biom15050650 40427543 PMC12109227

[50] Junka A Bartoszewicz M Smutnicka D Secewicz A Szymczyk P. Efficacy of antiseptics containing povidone-iodine, octenidine dihydrochloride and ethacridine lactate against biofilm formed by Pseudomonas aeruginosa and Staphylococcus aureus measured with the novel biofilm-oriented antiseptics test. Int Wound J. (2014) 11:730–4. 10.1111/iwj.12057 23445335 PMC7950748

[51] Falanga V Eaglstein WH Bucalo B Katz MH Harris B Carson P. Topical use of human recombinant epidermal growth factor (h-EGF) in venous ulcers. J Dermatol Surg Oncol. (1992) 18(7):604–6. 10.1111/j.1524-4725.1992.tb03514.x 1624634

[52] Tirma González-Mateo G Kopytina V, editors. Wound Healing - Mechanisms and Pharmacological Interventions. IntechOpen. Madrid: SEMERGEN (2025). 10.5772/intechopen.1008032

[53] Gisby J Bryant J. Efficacy of a new cream formulation of mupirocin: comparison with oral and topical agents in experimental skin infections. Antimicrob Agents Chemother. (2000) 44(2):255–60. 10.1128/AAC.44.2.255-260.2000 10639346 PMC89667

[54] Gottrup F. Oxygen in wound healing and infection. World J Surg. (2004) 28(3):312–5. 10.1007/s00268-003-7398-5 14961190

[55] Bullough WS Johson M. Epidermal mitotic activity and oxygen tension. Nature. (1951) 167:488. 10.1038/167488a0

[56] Horikoshi T Balin AK Carter DM. Effect of oxygen on the growth of human epidermal keratinocytes. J Invest Dermatol. (1986) 86:424–7. 10.1111/1523-1747.ep12285695 2427617

[57] Karasek MA. *In vitro* culture of human skin epithelial cells. J Invest Dermatol. (1966) 47:533–40. 10.1038/jid.1966.182 5957923

[58] Dimitrijevich SD Paranjape S Wilson JR Gracy RW Mills JG. Effect of hyperbaric oxygen on human skin cells in culture and human dermal and skin equivalents. Wound Rep Reg. (1999) 7(1):53–64. 10.1046/j.1524-475x.1999.00053.x 10231506

